# Novel Functional Glass–Ceramic Coatings on Titanium Substrates from Glass Powders and Reactive Silicone Binders

**DOI:** 10.3390/polym14194016

**Published:** 2022-09-26

**Authors:** Hamada Elsayed, Rachele Bertolini, Lisa Biasetto, Paulina Ożóg, Jozef Kraxner, Dušan Galusek, Enrico Bernardo

**Affiliations:** 1 Department of Industrial Engineering, Università Degli Studi di Padova, 35131 Padova, Italy; 2 Refractories, Ceramics and Building Materials Department, National Research Centre, Cairo P.O. Box 12622, Egypt; 3 Department of Management and Engineering, Università Degli Studi di Padova, 35122 Padova, Italy; 4 Centre for Functional and Surface Functionalized Glass (FunGlass), Alexander Dubček University of Trenčín, 911 01 Trenčín, Slovakia; 5 Joint Glass Centre of the IIC SAS, TnUAD and FChFT STU, 911 50, Trenčín, Slovakia

**Keywords:** polymer-derived ceramics, coatings, glass–ceramics, sinter–crystallization, titanium

## Abstract

‘Silica-defective glasses’, combined with a silicone binder, have been already shown as a promising solution for the manufacturing of glass–ceramics with complex geometries. A fundamental advantage is the fact that, after holding glass powders together from room temperature up to the firing temperature, the binder does not completely disappear. More precisely, it converts into silica when heat-treated in air. A specified ‘target’ glass–ceramic formulation results from the interaction between glass powders and the binder-derived silica. The present paper is dedicated to the extension of the approach to the coating of titanium substrates (to be used for dental and orthopedic applications), with a bioactive wollastonite–diopside glass–ceramic layer, by the simple airbrushing of suspensions of glass powders in alcoholic silicone solutions. The interaction between glass and silica from the decomposition of the binder led to crack-free glass–ceramic coatings, upon firing in air; in argon, the glass/silicone mixtures yielded novel composite coatings, embedding pyrolytic carbon. The latter phase enabled the absorption of infrared radiation from the coating, which is useful for disinfection purposes.

## 1. Introduction

Silicones, i.e., preceramic polymers yielding a silica-based residue upon thermal treatment [1], represent the starting point for a number of silicate ceramics [2]. The key advantages are the easiness in shaping the components using the application of polymer-based technologies, including additive manufacturing techniques, before thermal treatment, and high reactivity. The amorphous silica-based residue interacts with several metal oxide inclusions formed by the decomposition of mineral micro- and nanosized fillers (such as carbonates and hydroxides), yielding a target high phase purity silicate at a relatively low firing temperature [2]. Selected fillers, such as alkali borates and phosphates, may enhance the reactivity by forming a liquid phase upon firing and extend the approach beyond crystalline silicates. The borate and phosphate liquid phase (melt) turns into glass upon cooling, with the definition of a new range of ‘polymer-derived glass–ceramics’ [3,4].

Despite being mostly exploited for highly porous components, such as foams and scaffolds, silicone/filler mixtures may also be applied to the manufacturing of coatings, e.g., to prevent the oxidation of SiC porous bodies [5] or to enhance the integration of Ti implants in bone tissue applications [6]. In both cases, the approach is particularly attractive for its simplicity, since the fillers may be easily suspended in the alcoholic solutions of commercial silicones (with high silica yield upon firing), leading to slurries that can be used for dip coating or airbrush spraying. Upon solvent evaporation, silicones bind the filler particles up to the temperature of conversion into the desired silicate phase(s).

Airbrush-processed polymer-derived sphene coatings [6] demonstrated an inherent weakness of the approach. The gas release and the volumetric changes arising from the thermal transformation of both the silicone matrices and fillers may compromise the integrity of the coatings. The reaction may take place closer to the metallic substrate and later than at the surface. Such a difference may lead to thermal stresses and in turn, favor the formation of cracks. A simple remedy is represented by ‘dilution’, i.e., by the introduction of particles of the desired ceramic phases added as fillers [6]. This strategy substantially replicates the classical approach to polymer-derived ceramics proposed by Greil and co-workers [7], who defined both ‘active fillers’ (reacting with the ceramic residue of preceramic polymers and/or with the atmosphere) and ‘passive fillers’ (not reactive).

An alternative and less straightforward form of oxide fillers is represented by glass particles. Even if not contributing to the formulation of the final ceramic as ‘chemically active’ fillers, glass particles may be ‘physically active’: they form a liquid phase upon softening, sealing cracks and relaxing stresses [8,9].

Some research efforts have specifically concerned glasses as ‘integrative’ precursors of polymer-derived silicate ceramics. In their pioneering work, Ohl et al. [10] used borosilicate glass (commercial Duran^®^ glass) powders bound by a silicone polymer and including borax as a reactive filler. Na_2_O and B_2_O_3_ from the borax reacted with the amorphous silica from the silicone oxidation, yielding additional borosilicate glass. In other words, the ceramic product of the silicone/borax interaction was engineered to facilitate the full integration of Duran glass powder and the binder. Such integration is the basis of studies on bioactive wollastonite–diopside glass–ceramics, involving both interactions between silicones and CaO- and MgO-based fillers and the sinter–crystallization of CaO-MgO-SiO_2_ glass powders. Before crystallizing, softened glass produces a liquid phase without altering the overall chemical composition as could occur with borate and phosphate fillers [4].

A more refined concept involves glasses specifically designed to yield glass–ceramics after interactions with silicone binders and explored for the manufacturing of glass–ceramic joints for planar solid oxide fuel cell (SOFC) designs [11] and glass–ceramic scaffolds for bone tissue engineering, prepared both by direct ink writing and stereolithography [12,13]. Glass–ceramics were derived from the sinter–crystallization of specified powdered parent glasses or from the interaction between silicones and powdered ‘silica-defective glasses’. The latter were designed to include oxides in the same proportions as in the parent glass, except for silica: the exact chemical composition of parent glass was achieved by mixing with silica originating from the oxidation of silicone binders. Instead of ‘conventional’ sinter–crystallization (sintering with concurrent crystallization), silica-defective glasses combined with silicones experience a form of ‘reactive’ sintering, with the final glass–ceramic not relying on a simple physical transformation but on a chemical reaction [14]. The phase assemblage of the final glass–ceramic, passing from sinter–crystallization to reactive sintering, is not substantially altered [11,12,13].

A key motivation for reactive sintering with silicone and silica-defective glasses is the control of the quality of the final glass–ceramic products. Glass–ceramic joints in SOFCs typically derive from the sandwiching of glass powders between a ceramic electrolyte and a metal substrate. Glass powders along with organic binders are deposited by screen-printing [11]. On the other hand, glass–ceramic scaffolds may be formed by the extrusion or photocuring of glass powders, which are also mixed with organic binders [12,13]. In all cases, the decomposition of organic additives at the early stages of heat treatment (at temperatures below the glass transition temperature, T_g_), leaves glass powders ‘unbound’ well before viscous flow sintering takes place (at temperatures above T_g_). Owing to the limited packing of powders, de-bonded parts are extremely fragile, and any form of mechanical stress may lead to their failure. Silicones represent advanced binders, since their degradation is not complete, and up to high temperatures, the glass powders remain bound by the ceramic residue of the binder [11,12,13].

The present paper aims to combine the concept of ‘reaction–sintered’ glass–ceramics with that of airbrush-processed, silicone-derived silicate coatings on Ti substrates [6,14,15]. Powdered silica-defective glasses constitute the only filler for a commercial silicone polymer, not considered merely as a silica source. In addition to yielding homogeneous, crack-free coatings, the approach yielded a nanocomposite after firing of the silicone/glass mixture in an argon atmosphere. Firing in an Ar atmosphere led to the conversion of the silicone binder into silica (reacting with silica-defective glasses) and pyrolytic carbon, thus imparting the coatings with additional functionality.

## 2. Materials and Methods

### 2.1. Glass Synthesis and Preliminary Studies on Glass–Ceramic Pellets

Table 1 reports the chemical composition of a reference glass [4] yielding upon heat treatment a wollastonite–diopside glass–ceramic, named RG, and of two ‘silica-defective’ variants, V1 and V2. The glasses were produced by melting raw materials (carbonates and oxides) in a furnace at 1300 °C for 1 h in a Pt-Rh crucible. The melts were quenched by direct pouring them onto a cold metal plate, obtaining coarse fragments. The fragments were subjected to scanning electron microscopy (JFM 7600, Jeol Ltd., Akishima, Tokyo, Japan), equipped with energy dispersive spectroscopy (EDS, Oxford, UK) in order to verify the chemical composition.

The glass fragments were ball milled (Pulverisette 6, Fritsch GmbH, Idar-Oberstein, Germany) and manually sieved to produce fine powders. Only the particles with a diameter below 75 µm were used for further work. The particle size distribution (also shown in Table 1) was assessed by means of laser light scattering (Mastersizer Hydro 2000, Malvern Panalytical, Malvern, UK) on powders dispersed in distilled water.

Monolithic pellets were prepared by mixing glass particles with a commercially available silicone H44 (Wacker-Chemie GmbH, Munich, Germany). The polymer was dissolved in isopropanol (15 mL for 10 g of final ceramic) and then mixed with glass powders in four silicone–glass ratios (V1A, V1N, V2A, and V2N), as reported in Table 1. The mixing was performed under magnetic stirring, followed by sonication for 10 min, leading to stable and homogeneous dispersions. The mixtures were poured into PTFE containers and dried at 80 °C overnight. After drying, the silicone-based mixtures formed solid fragments, which were then pulverized (<100 µm) by ball milling at 350 rpm for 30 min.

Composite silicone–glass powders were cold-pressed in cylindrical steel die applying a pressure of 20 MPa for 1 min, without any additive. Pellets with a diameter of approximately 17 mm and 2 mm thick were obtained. For comparison, pellets were also prepared from powders of the reference glass. The specimens were heat-treated, using the heating rate of 5 °C/min, up to 900 °C for 1 h.

Powdered pellets were analyzed by X-ray powder diffraction with Bragg–Brentano configuration (XRD, Bruker AXS D8 Advance, Karlsruhe, Germany). A semiautomatic phase identification was conducted using the Match! program package (Crystal Impact GbR, Bonn, Germany), supported by data from PDF-2 database (ICDD-International Centre for Diffraction Data, Newtown Square, PA, USA).

### 2.2. Airbrush Coating

**Figure 1:**
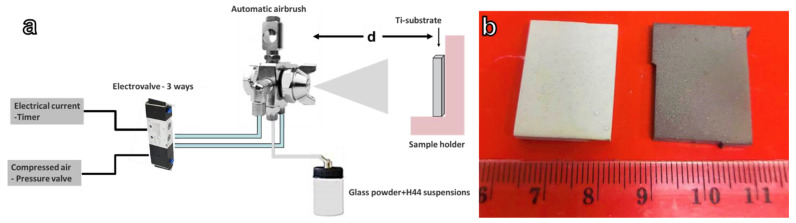
(**a**) Scheme of coating apparatus; (**b**) examples of coated Ti blocks after heat-treatment at 900 °C; on the **right**: sample heated-treated in the air, on the **left**: sample heated-treated in argon.

Two suspensions of H44 with the same solid content of ≈40 vol% of glass powder were prepared. The homogenous suspensions were homogenized using a planetary mixer (Thinky Are-250, Intertronics, Kidlington, UK), at a speed of 2000 rpm for 5 min. Commercially pure grade titanium (Grade 1 Titanium, Torresin Titanio s.r.l., Padova, Italy) substrates with the dimensions of 20 × 15 × 3 mm^3^ were prepared. The coating process was performed using a commercially available automatic airbrush (Prona-RA-C2, Prona Tools, Toronto, ON, Canada M3J 3A1), as outlined in Figure 1a. The air pressure was fixed at 3 bar, the substrate–nozzle distance (d) at 20 cm, and the nozzle opening was set at a diameter of 1 mm. The distance of 20 cm was selected after preliminary optimization tests. The coating time varied from 1.5 to 3 s to study its effect on coating properties. After the coating, the coated substrates were then heat-treated in air or argon atmosphere at 900 °C for 1 h, using a heating rate of 5 °C/min. The heat treatment led to homogeneous, adherent, and crack-free coatings, as illustrated in Figure 1b. The different firing atmospheres resulted in different coloration. (Samples fired in air were white, and samples fired in argon were dark grey.) All products were characterized by X-ray diffraction (Bruker AXS D8 Advance, Karlsruhe, Germany), followed by semiautomatic phase identification, as reported above.

### 2.3. Surface Finish Assessment

The morphology of the coatings was examined by field emission gun scanning electron microscopy (FEG-SEM, Quanta 250 Fei, Eindhoven, The Netherlands) and energy dispersive spectroscopy (EDS, EDAX AMETEK, Inc., Mahwah, NJ, USA). The investigations were performed on both cross-section and surface of the coated samples. The sections of the samples were first embedded into a resin, ground up to 2100 SiC grit paper and finally polished using a 3 μm diamond paper and a SiO_2_ colloidal dispersion in demineralized water and H_2_O_2_. The polished samples were ultrasonically cleaned in ethanol for 15 min, rinsed in demineralized water, and finally dried by compressed air. EDS line scans of scratched tracks and EDS maps (surface) were carried out to better understand the crack mode and coating composition.

**Figure 2:**
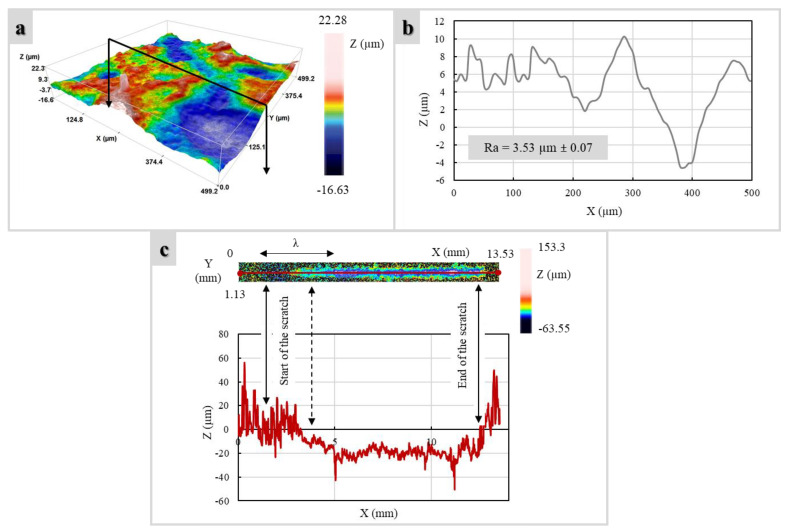
(**a**) 3D topography and (**b**) 2D corresponding profile of surface relative to the received substrate topography; (**c**) 3D topography and corresponding 2D profile of the scratch track of the V1A (1.5 s) sample.

The Sensofar Plu Neox (Sensofar, Barcelona, Spain) confocal microscope with a 20 × objective was used to acquire the surface topography of both the prepared glass coatings and the substrate. The acquired height maps with lateral dimensions of approximately 15.1 mm × 0.72 mm (see an example of the cropped surface as shown in Figure 2a) underwent form removal by subtraction of the mean plane. For each measured topography, at least three surface profiles were extracted to compute the roughness parameters according to ISO 4288 standard, with λ_s_ filter equal to 8 μm, cut-off λ_c_ equal to 2.5 mm, and sampling length of 12.5 mm (see example in Figure 2b). The arithmetic mean deviation of the assessed profile (Ra) was considered as the most common parameter used in the literature [16].

### 2.4. Mechanical Characterization: Scratch Test

To estimate the adhesion strength of prepared coatings, scratch tests were performed using a Bruker-UMT-3™ tribometer (Bruker, Karlsruhe, Germany). A triangular composite diamond tip with a radius of 800 µm was used as a scratching tool. During the test, the scratch tip was moved at a constant speed of 0.5 mm/sec along the sample surface under a load which increased incrementally from 1 N to 10 N. The scratch tracks were 10 mm long. The test was performed three times to verify repeatability of the measurement.

To identify the critical load at which the coating fails L_c_ (N), the scratch tracks were examined using an optical profiler under focus variation mode. After each test, an area of 13.53 mm × 1.3 mm was scanned, as reported in Figure 2c. After that, the profile in correspondence of the scratch width center and whole length was extracted. The distance used to compute L_c_ was equal to the length between the scratching starting point and the first X-coordinate at which the Ti substrate was reached by the scratching tool tip, i.e., the value of the highest depth (Z) coordinate.

### 2.5. Infrared Heating Test

Selected samples were heated by a commercial incandescent IR lamp (Philips R95, Eindhoven, The Netherlands). The lamp was placed at a distance of 100 mm over the samples supported by a refractory block. The whole apparatus was covered by a cardboard box. The IR lamp was kept on for 120 s, after which the cardboard box was removed.

An infrared camera (FLIR™ A6000 Series, Teledyne FLIR LLC, Wilsonville, OR, USA) was used to record the temperature of the samples by applying an emissivity value of 0.9 in accordance with [17].

## 3. Results

### 3.1. Phase Development in Glass–Ceramic Pellets and Coatings

**Figure 3:**
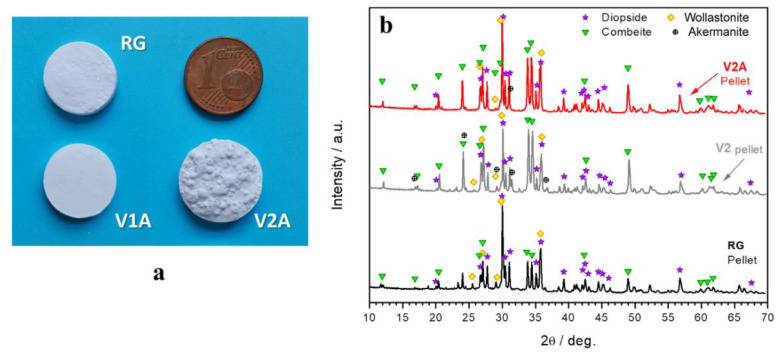
(**a**) Examples of glass–ceramic pellets (fired in air); (**b**) comparison between sinter–crystallization of reference glass, sinter–crystallization of V2 glass, and H44 silicone–V2 reaction.

Figure 3a shows some examples of glass–ceramic pellets after firing at 900 °C in air. Pressed powders of the reference glass (RG) turned into dense pellets with a total uniform shrinkage of ≈10–12%. Similar pellets resulted from the firing of the mixture based on the V1 glass (lower silica reduction) with the addition of the H44 silicone binder. The V2 (higher silica reduction) mixed with more H44 led to foamed samples. A different morphology of pellets did not correspond to marked differences in the overall phase assemblage. Figure 3b illustrates the ‘more silica-defective’ glass: the samples from the firing of RG, V2 alone, and the V2A mixture all featured diopside (CaMgSi_2_O_6_, PDF#72-1497), wollastonite (CaSiO_3_, PDF#84-0655), and combeite (Na_2_Ca_2_Si_3_O_9_, PDF#75-1687). In V2, without silicone, the crystallization of combeite was much more pronounced than in the reference glass, and traces of åkermanite (Ca_2_MgSi_2_O_7_, PDF#83-1815) were detected. In contrast, the pattern of the V2A sample did not feature any extra phase and presented the main diffraction line intensities of combeite well below the main diffraction line intensities of diopside and wollastonite (2θ~30°), similar to the XRD pattern of the RG pellet.

**Figure 4:**
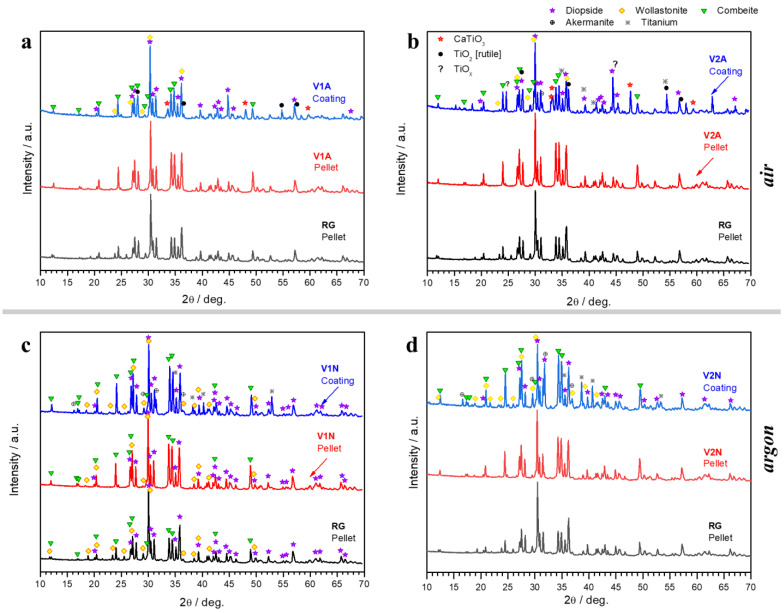
Comparison of XRD patterns of glass–ceramic coatings (deposition time: 1.5 s) from V1 and V2 glasses reacting with H44 silicone in air (**a**,**b**) and in argon atmosphere (**c**,**d**), compared with pellets fired under the same conditions.

The phase assemblage of the sinter–crystallized RG and the products of the reaction of the H44 silicone with the V1 and V2 glasses were even more similar when switching from pellets to coatings, at least under some specific conditions, as illustrated in Figure 4.

The intensity of the diffraction maxima of sinter–crystallized RG and V1A coatings were nearly identical (Figure 4a). The same can be observed for the glass–ceramic coating prepared from the V2A formulation (with more silica from the binder), despite the differences in the appearance of the pellet (Figure 4b). Some extra phases could be attributed to the metallic substrate that oxidized during the heat treatment, leading to formation of titanium oxides (rutile, TiO_2_, PDF#78-1510, and TiO_x_, PDF#85-2084). The oxides reacted with the CaO from the glass, forming CaTiO_3_ (PDF#88-0790). Traces of åkermanite are also likely to be present.

The content of extra phases was minimized when using less ‘silica-defective’ V1 glass and firing in argon (V1N, Figure 4c). In this atmosphere, H44 did not simply convert to silica. With the exception of more intense combeite peaks, the glass–ceramic coatings were similar to the sinter–crystallized RG composition (Figure 4c,d).

### 3.2. Morphology of Coatings

**Figure 5:**
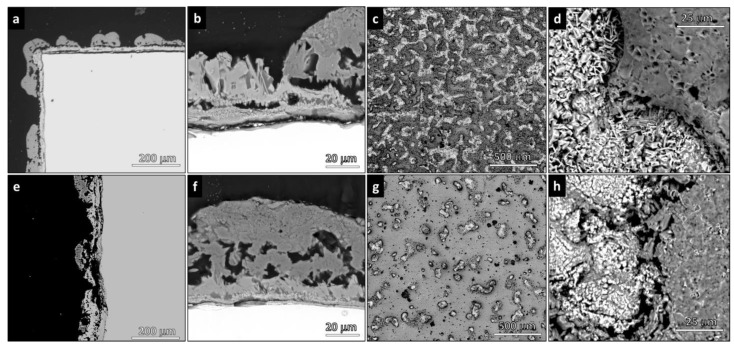
Microstructural details of cross-section and surface of V2A glass–ceramic coatings: (**a**–**d**) 1.5 s; (**e**–**h**) 3 s.

The micrographs in Figure 5a,b and Figure 5e,f display the cross-section of V2A coatings processed at 1.5 and 3 s, respectively. Figure 5c,d,g,f refer to surfaces. It was observed that with increasing spraying time, the total area of the Ti-rich zones (lighter areas) decreased. These areas, however, did not correspond to uncoated metal. As shown in Figure 5a, the metallic surface was covered by an oxide scale; glass–ceramic droplets were attached on the top of this oxide layer. The TiO_2_ layer was less visible for longer spraying time (Figure 5e,f). The glass–ceramic deposits were internally porous (Figure 5b,f).

**Figure 6:**
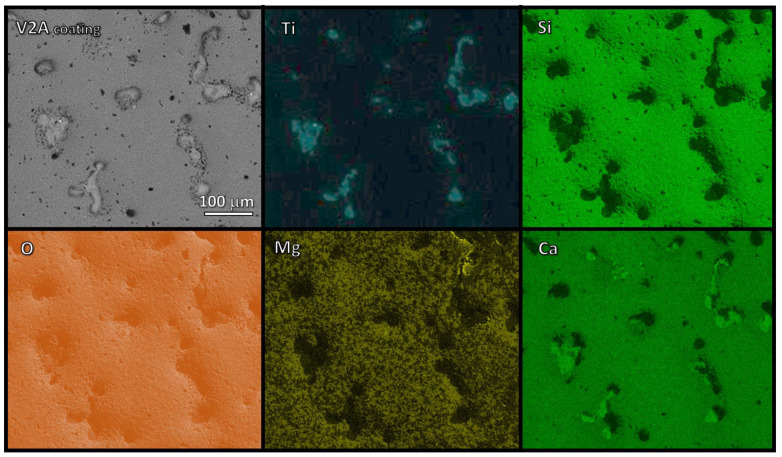
EDS maps of V2A (1.5 s) glass–ceramic coating.

The EDS maps of the V2A (1.5 s) glass–ceramic coatings (Figure 6) confirmed the presence of areas that were not covered by glass–ceramic material so that the fluorescence signal of titanium was not masked. The characteristic elements (Ca, Mg, Si) of the glass–ceramic coating were all uniformly distributed but with one fundamental difference: while magnesium and silicon remained concentrated in the glass–ceramic material, some calcium could also be found in zones with high titanium concentration. The results support the XRD data, which documented the presence of perovskite (CaTiO_3_) diffraction maxima (Figure 4b).

**Figure 7:**
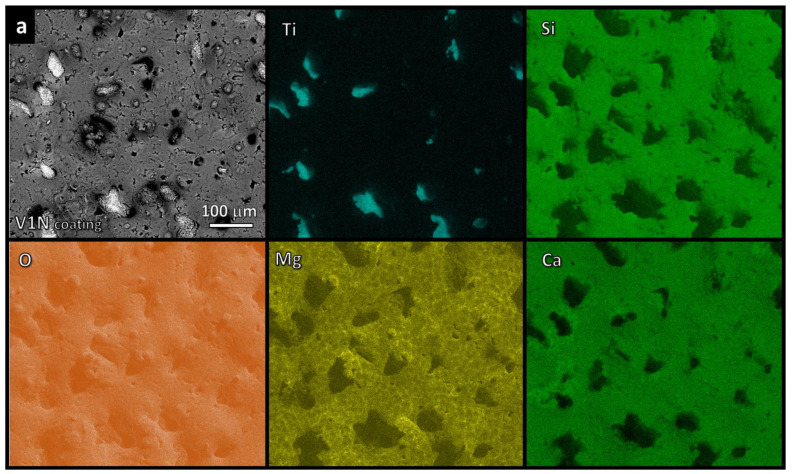

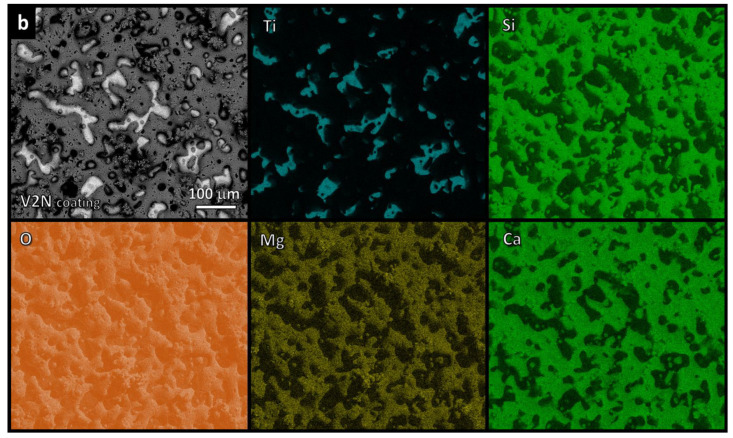
EDS maps of glass–ceramic coatings fired in argon: (**a**) V1N (1.5 s) (**b**) V2N (1.5 s).

A clear separation of the elements between coated and uncoated zones was characteristic for the glass–ceramic coatings fired in argon, irrespective of the amount of silicone (less silicone in V1N, Figure 7a; more silicone in V2N, Figure 7b). Uncoated zones contained titanium. Coated zones contained all other elements (Si, Mg, Ca, and O). The maps of Ca and Si were nearly identical.

**Figure 8:**
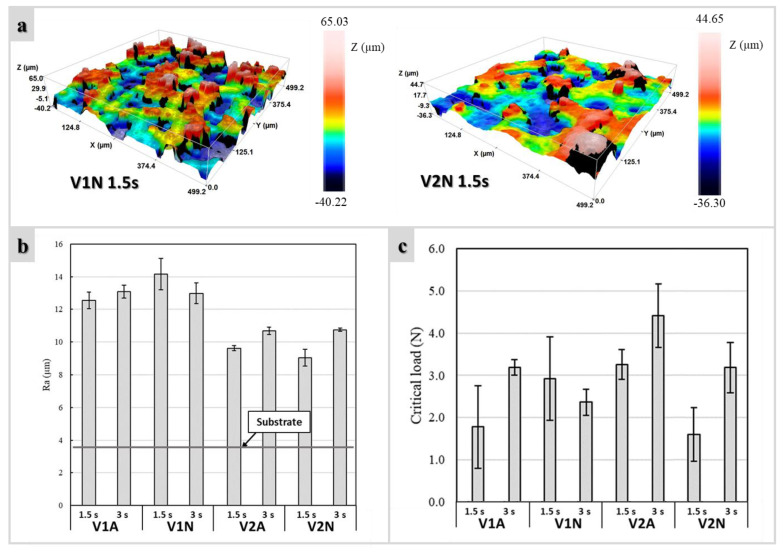
(**a**) 3D topography of argon-treated samples; (**b**) surface roughness measurements at varying processing parameters; (**c**) critical load at varying processing parameters.

The presence of the uncoated zones resulted in a dramatic increase (>200%) of surface roughness, passing from the starting substrate (Figure 2a) to materials with glass–ceramic cladding (Figure 8a). It was noticed that the adoption of the V1 glass as a precursor led to rougher surfaces compared to the V2. No clear influence of the deposition time and atmosphere on Ra values was detected, with comparable surface roughness profiles for the same used glass (Figure 8b).

### 3.3. Scratch Tests

Figure 8c shows the critical load as a function of the process parameters. In general, longer deposition times (3 s) enhanced the strength/adherence. The strongest coatings were prepared from the V2 combined with H44 and fired in air (see V2A (3 s)), but quite good results were also achieved by firing in argon (V1N (1.5 s) and V2N (3 s)).

**Figure 9:**
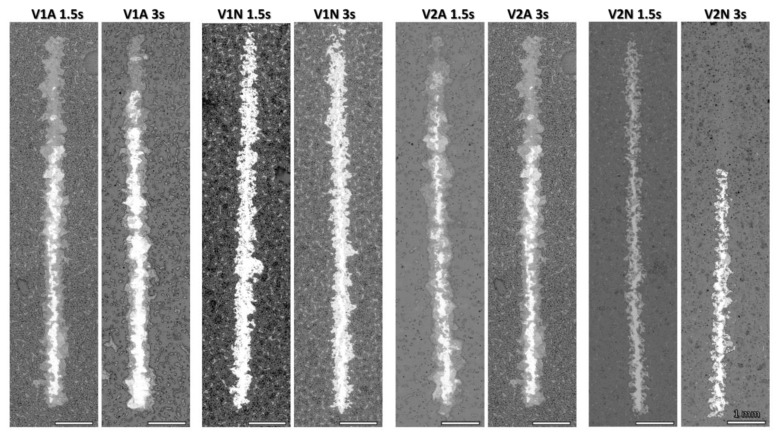
Scratch lines of the coatings deposited for 1.5 s and 3 s.

Interesting differences emerged from the SEM examination of the scratch tracks (Figure 9). Gradual flaking was observed in the samples processed in the air, while a detached coating was found in the samples of identical composition fired in argon. Along with a mite rupture, heat treatment in the air provoked extensive detachment of the coating compared to the samples heat-treated in argon, which, in contrast, presented ruptures characterized by a reduced size.

### 3.4. Infrared Heating

**Figure 10:**
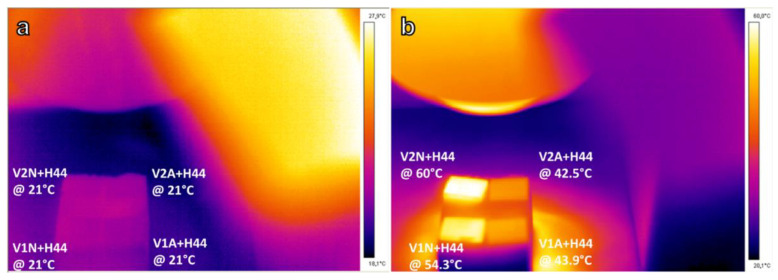
Infrared image of coated Ti blocks: (**a**) before, and (**b**) after 120 s IR irradiation.

Changing the atmosphere from air to argon resulted in a darker coloration of the coating (Figure 1b), attributed to the conversion of H44 into silica and pyrolytic carbon. The presence of the carbon phase was confirmed by the sensitivity of the coated samples to infrared radiation (Figure 10). The temperature of coated Ti blocks fired in argon significantly increased after IR irradiation, reaching a temperature of ~60 °C, which was significantly higher than in the analogous blocks processed in air.

## 4. Discussion

### 4.1. Phase Evolution

The main findings of this work are represented by the flexibility of the silicone/glass interaction, which led to glass–ceramic coatings with a phase assemblage resembling that of the reference system (sinter–crystallized RG), operating with both silica-defective glasses and firing atmospheres. The final products resulted from the effective interaction between polymer-derived silica and glass fillers. This was confirmed by the EDS mapping of silicon (Figure 6 and Figure 7) showing comparable intensities of signals for Si, calcium, and/or magnesium in all characterized areas. In other words, the ceramic residue of the polymer effectively mixed with the glass, yielding the desired glass–ceramic system. It should be noted that the comparison between sinter–crystallized RG and products from silicone/glass interaction was not affected by granulometry: as reported in Table 1, all glass powders showed a similar particle size distribution.

Treatment of the less silica-defective glass (V1A) in the air represented the ‘easiest’ option. To achieve the overall chemical composition of RG, the lowest addition of H44 to V1 was needed (see Table 1), which minimized the amount of gaseous byproducts of silicone decomposition released upon firing. For V2, the preparation of dense pellets was complicated, since the higher amount of binder led to the enhanced release of gases into a mass of softened glass with a low silica content and hence, a lower viscosity. This resulted in foaming of the glass melt (Figure 3a). However, X-ray powder diffraction analysis confirmed the occurrence of the reaction between glass and silicone residue (Figure 3b): while V2 alone yielded åkermanite as a relatively silica-poorer alternative to diopside and wollastonite and featured the precipitation of combeite, the H44/V2 mixture (V2A) yielded three crystalline phases (diopside, wollastonite, and combeite) in ratios similar to crystallized RG.

The increase in the combeite content together with the simultaneous reduction in the intensity of the wollastonite-related diffraction maxima can be justified by the catalytic effect of the multiple interfaces between the softened V2 glass and the H44-derived silica, combined with the inherently higher mobility of alkali ions.

Due to their reduced thickness, the release of gaseous moieties was not an issue for the coatings. Silica-defective glasses combined with H44 led to glass–ceramic ‘droplets’ on the metallic substrate with some internal pores, but no foaming was observed (see Figure 6). As shown in Figure 4a,b, the phase assemblage of the glass–ceramic coatings treated in air was very similar to that of the crystallized RG. The only exception was the formation of CaTiO_3_, resulting from the interdiffusion between the glass and titanium dioxide.

The treatment in argon prevented the substrate from oxidating. No carbide phase(s) appeared as might be anticipated from reactions between the metallic substrate and C-rich moieties released from H44 or from the phase separation of the oxycarbide (SiOC) ceramic residue of the polymer. According to Scheffler et al., the residue of H44 ceramization in a nonoxidizing atmosphere consists of 18.7%Si-28.7%O-52.6%C (at%) or (Si_3_O_4.6_C_8.45_) [18], but it does not represent a single phase. Instead, turbostratic carbon nanosheets are dispersed in a silica-based glass, featuring Si-C bonds. Silicon carbide may separate from the glass [19], and the composition of H44-derived SiOC can be expressed as follows:

Si_3_O_4. 6_C_8.45_ ↔ 2.3 SiO_2_ + 0.7 SiC + 7.75 C

While V1A and V2A were formulated according to the reaction of glasses with the full residue of H44 (with a yield of 52.5 wt.%) [20] consisting of pure silica, V1N and V2N were based on the assumption of glasses combining only with the silica fraction. SiC and pyrolytic carbon were considered as additional phases. However, the absence of SiC traces (even for V2N featuring the highest amount of H44) and the close match of Si maps with those of Ca and Mg suggest a different evolution. H44 transformed into a mixture of silica (mixed with CaO, MgO, and Na_2_O mostly bound in crystalline silicates) and pyrolytic carbon.

**Figure 11:**
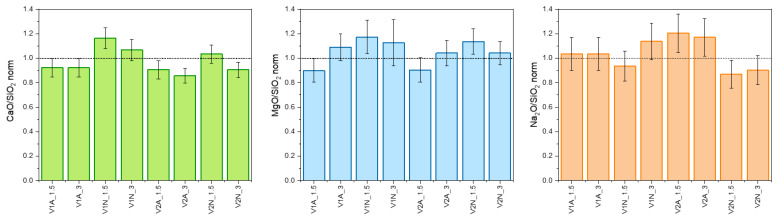
Normalized CaO/SiO_2_, MgO/SiO_2_, and Na_2_O/SiO_2_ ratios inferred from EDS analysis on glass–ceramic coatings.

The increased intensity of the combeite-related diffraction maxima (Figure 4c,d) could suggest an enhanced silica yield for treatments in argon. However, this was not confirmed by the quantitative EDS analysis (from areas such as those shown in Figure 6 and Figure 7). Figure 11, in particular, shows CaO/SiO_2_, MgO/SiO_2_, and Na_2_O/SiO_2_ ratios inferred from the elemental analysis (expressed in oxides) on all coatings, normalized on the same ratios calculated from a RG glass–ceramic pellet. The data feature a large scattering but confirm the similarity between coatings (from glass/silicone interaction) and the reference glass–ceramic.

In conclusion, when reacting with fillers, the ceramic yield from silicones is hardly predictable in terms of phases and quantities and deserves additional studies. Deviations from the theoretical yield may also justify unexpected crystal phases (e.g., åkermanite from the characteristic peak at 2θ~31.3° in the V2N coating).

### 4.2. Roughness, Scratch Resistance, and Functionalization

In general, a good match with the reference glass–ceramics was achieved for both silica-defective glasses treated in air (V1A, V2A) and V1 treated in argon (V1N). The approach, however, was not aimed at just the preparation of glass–ceramic coatings with specified composition and phase assemblage. The methodology is intended to facilitate the tuning of some of its functionalities. In particular, the roughness of coatings is known to modulate the adhesion of titanium implants to bone tissue [21,22].

The deposition of silicone/glass droplets leaving some uncoated metal was intentionally carried out to favor the formation of an abundant surface roughness. Obviously, the morphology of the final glass–ceramic coating was conditioned by the different formulations. The V2-derived coatings were smoother due to the enhanced viscous flow of a glass with a much reduced silica content.

In Table 2, critical loads (Lc) for different ceramic coatings on Ti substrates are summarized. In general, the critical load values were lower than those experienced with other polymer-derived coatings deposited by airbrushing [15], but they remained within the range reported for many ceramic coatings on Ti substrates, such as sol-gel-derived sphene [23] and hydroxyapatite (HAp) [24,25]. Treatments in argon showed a multiform effect: the clear detachment may be attributed to a weaker interface with the metal substrate (in the absence of a titanium oxide and CaTiO_3_). The reduction in lateral cracking may be due to the presence of the internal interfaces between silicate phases and pyrolytic carbon: the machinability of engineering ceramics, in fact, is typically improved by the introduction of weaker secondary phases, such as mica, hexagonal boron nitride, and graphite [26]. Along with the roughness determined by the airbrush deposition technique, the interaction of bone tissue with Ti pieces coated by the glass–ceramic/carbon composites could be favored by suitable post-firing machining.

The secondary carbon phase, in addition to providing a ‘graceful’ scratching, was particularly useful for achieving the photothermal effect. In good agreement with recent findings on polymer-derived silicate biomaterials [30,31,32], the heating of the samples under a simple IR lamp was very rapid and intensive, far exceeding the threshold (55 °C) required for disinfection by the thermal destruction of bacteria [33].

## 5. Conclusions

Silicones are confirmed in their role as advanced binders for the development of glass–ceramic products, starting from fine glass powders. Starting from different silicone/glass proportions, the interaction between a commercial silicone and glass powders yields nearly the same ‘target’ glass–ceramic system through the proper design of the chemical composition of the glass. The interaction is also verified for glass–ceramics in the form of coatings on a titanium substrate, which are easily produced using the airbrush deposition of silicone-based slurries. The change in the atmosphere during pyrolysis from air to argon, performed after the adequate optimization of the silicone/glass ratios, appears to be a simple tool for the preparation of composite coatings, comprising ‘in situ’ formed pyrolytic carbon. The presence of residual carbon adds interesting functionalities to the coatings, such as machinability and sensitivity to fast heating by IR irradiation.

## Figures and Tables

**Table 1:** Chemical composition of the studied glasses and glass–silicone ratios.

	RG	Silica-Defective Glasses	
		V1 (Low Silica Reduction)	V2 (High Silica Reduction)
*Chemical composition (wt%)*			
SiO_2_	58.8	55.5	51.6
MgO	8.6	9.3	10.1
CaO	21.8	23.6	25.7
Na_2_O	9.9	10.6	11.5
Li_2_O	0.9	1.0	1.1
*Particle size distribution*			
d_10_ (µm)	3.7	3.8	4.3
d_50_ (µm)	11.3	12.0	11.8
d_90_ (µm)	29.2	32.7	30.9
*Silicone–glass ratios (wt%)*			
Firing in air		13.5–86.5 (V1A)	25–75 (V2A)
Firing in argon		17.5–82.5 (V1N)	31–69 (V2N)

**Table 2:** Overview of critical load Lc for bioglasses and bioceramic coatings deposited on Ti substrates.

Sample	Lc [N]	Deposition Method	Reference
V1A-V2A	<5	Airbrush	This work
V1N-V2N	<4.5	Airbrush	This work
45S5 and CaK	0.8–4.5	Pulsed electron deposition	[27]
45S5	18–21	High velocity suspension flame spraying	[28]
Sphene	<1	Sol-gel	[23]
	5.6–8.6	Airbrush	[15,29]
HAp	11	Plasma spray	[24]
	<2.5	Electrochemical process	[25]

## Data Availability

The data presented in this study are available on request from the corresponding author.
